# METFORMIN: an efficacy, safety and pharmacokinetic study on the short-term and long-term use in obese children and adolescents – study protocol of a randomized controlled study

**DOI:** 10.1186/1745-6215-15-207

**Published:** 2014-06-05

**Authors:** Marloes P van der Aa, Marieke AJ Elst, Edgar GAH van Mil, Catherijne AJ Knibbe, Marja MJ van der Vorst

**Affiliations:** 1 Department of Paediatrics, St. Antonius Hospital, Koekoekslaan 1, 3435 CM Nieuwegein, The Netherlands; 2 Department of Paediatrics, Jeroen Bosch Hospital, Henri Dunantstraat 1, 5223 GZ’s Hertogenbosch, The Netherlands; 3 Department of Clinical Pharmacy, St. Antonius Hospital, Koekoekslaan 1, 3435 CM Nieuwegein, The Netherlands

**Keywords:** Efficacy, Insulin resistance, Metformin, Paediatric obesity, Pharmacokinetics, Safety

## Abstract

**Background:**

The prevalence of childhood obesity and insulin resistance is rising, increasing the risk of diabetes mellitus type 2. To prevent these complications, lifestyle intervention is the corner stone in treatment. However, long-term efficacy of lifestyle intervention is questionable. In addition to lifestyle intervention, pharmacological treatments have been explored. Metformin has been shown to be moderately effective to reduce BMI in obese adolescents with hyperinsulinemia. However, data on pharmacokinetics and long-term efficacy and safety are lacking as well as an evidence-based dosing regimen for this age group. The primary objective of the METFORMIN study is to determine the effect of adding metformin treatment to lifestyle intervention in reducing BMI in obese adolescents with insulin resistance. In addition, the pharmacokinetics of metformin in obese adolescents will be studied.

**Methods/design:**

The METFORMIN study is a multi-centre prospective study that consists of two 18-month phases: a double-blind randomized placebo-controlled trial (part 1) and an open-label follow-up study (part 2). During part 1, the participants will be given metformin 1,000 mg or placebo twice daily and will be offered a lifestyle intervention programme; 144 participants will be included, 72 in each arm. Primary endpoints are reduction in body mass index, insulin resistance, and percentage body fat.

**Discussion:**

This study will provide data on short- and long-term efficacy and safety of metformin and on the pharmacokinetics of metformin in obese adolescents.

**Trial registration:**

ClinicalTrials.gov number NCT01487993; EudraCT nr. 2010-023980-17. Registration date: 06-01-2011

## Background

The prevalence of obesity in adolescents is increasing rapidly, having a significant impact on both physical and psychosocial health [1]. Currently, the worldwide prevalence of obesity in children and adolescents is 2% to 3%, using the International Obesity Taskforce standard definition for paediatric obesity in children and adolescents 5 to 17 years of age [2]. In the Netherlands, the prevalence of obesity in children and adolescents (4–15 years) of Dutch descent is 1.8% in boys and 2.2% in girls. In Turkish boys and girls, these numbers are higher, 8.4% and 8.0%, respectively [3].

In obese children and adolescents, insulin resistance, impaired fasting glucose, impaired glucose tolerance, dyslipidaemia, and hypertension occur with increased frequency [4-6]. In addition, several medical conditions, such as poor pulmonary function, hepatic steatosis, sleep apnoea, and orthopaedic complications, are associated with obesity [1,5,7,8]. These medical conditions often persist into adulthood and will result in substantial psychosocial and somatic morbidity, with loss of school or working days [9].

Current treatments for obesity are lifestyle, drug, and surgical interventions [10-12]. Behavioural lifestyle intervention can produce significant reduction of obesity in children and adolescents [13]. However, the efficacy of lifestyle intervention programs on body mass index (BMI) and all related complications on the long-term are questionable, taking into account the high drop out and the frequent relapse of obesity in this group of patients. Therefore, in clinical practice, adding a pharmacological agent to conventional treatment is often considered. Three agents have been studied: orlistat, a gastrointestinal lipase inhibitor, sibutramine, a serotonin and noradrenalin re-uptake inhibitor, and metformin, an insulin sensitizing agent [10-12,14]. Both orlistat and sibutramine have been shown to have an additional reducing effect on the absolute BMI in children and adolescents, yet medication-related adverse effects, such as tachycardia, hypertension, arrhythmia, and gastro-intestinal tract symptoms, were frequently reported [11,15,16]. Efficacy of metformin was investigated in hyperinsulinemic, obese adolescents by Park et al. in a systematic review [12]; they concluded that metformin is moderately efficacious in reducing BMI and insulin resistance in the short term (less than 6 months). The authors stated that large long-term studies are needed to establish the role of metformin in the treatment of obese adolescents. This conclusion is based on studies in obese adults without Type 2 diabetes mellitus (T2DM), in which metformin has been shown to prevent progression from impaired fasting glucose and impaired glucose tolerance to T2DM [17,18]. Although results in obese children and adolescents are sparse, these results, and the observed benefits in adults, have led to increased off-label use of metformin in obese children and adolescents with, and even without insulin resistance [19-26].

In conclusion, obesity in adolescence is increasing rapidly, with large medical and psychosocial sequelae. While standard treatment is lifestyle intervention, metformin is often added to this treatment, despite the lack of proper randomised trials on efficacy and safety, particularly upon prolonged treatment [12,27-31]. While metformin is licensed in adolescents for the treatment of T2DM, it is not yet licensed for obese children with and without insulin resistance.

## Methods/design

### Objectives

This study has four main objectives:

The primary objective of the METFORMIN study is to determine the efficacy of metformin in combination with lifestyle intervention in obese adolescents with insulin resistance versus placebo with lifestyle intervention after 18 months in reducing BMI and insulin resistance. The secondary objective is to determine the safety and tolerability of metformin in obese adolescents with insulin resistance. The tertiary objective is to study the population pharmacokinetics (PK) of metformin in obese adolescents. Finally, the quaternary objective is to determine the long-term (36 months) efficacy and long-term safety and tolerability of metformin in obese adolescents with insulin resistance.

Other objectives are to compare values of body fat measured using bio-impedance with values of body fat measured using dual energy X-ray absorptiometry (DEXA scan), and to compare insulin sensitivity measured by the Whole Body Insulin Sensitivity Index with insulin sensitivity calculated by Homeostasis Model Assessment for Insulin Resistance (HOMA-IR) in obese children and adolescents. Furthermore, arterial stiffness will be measured and evaluated over time.

### Design

**Figure 1:**
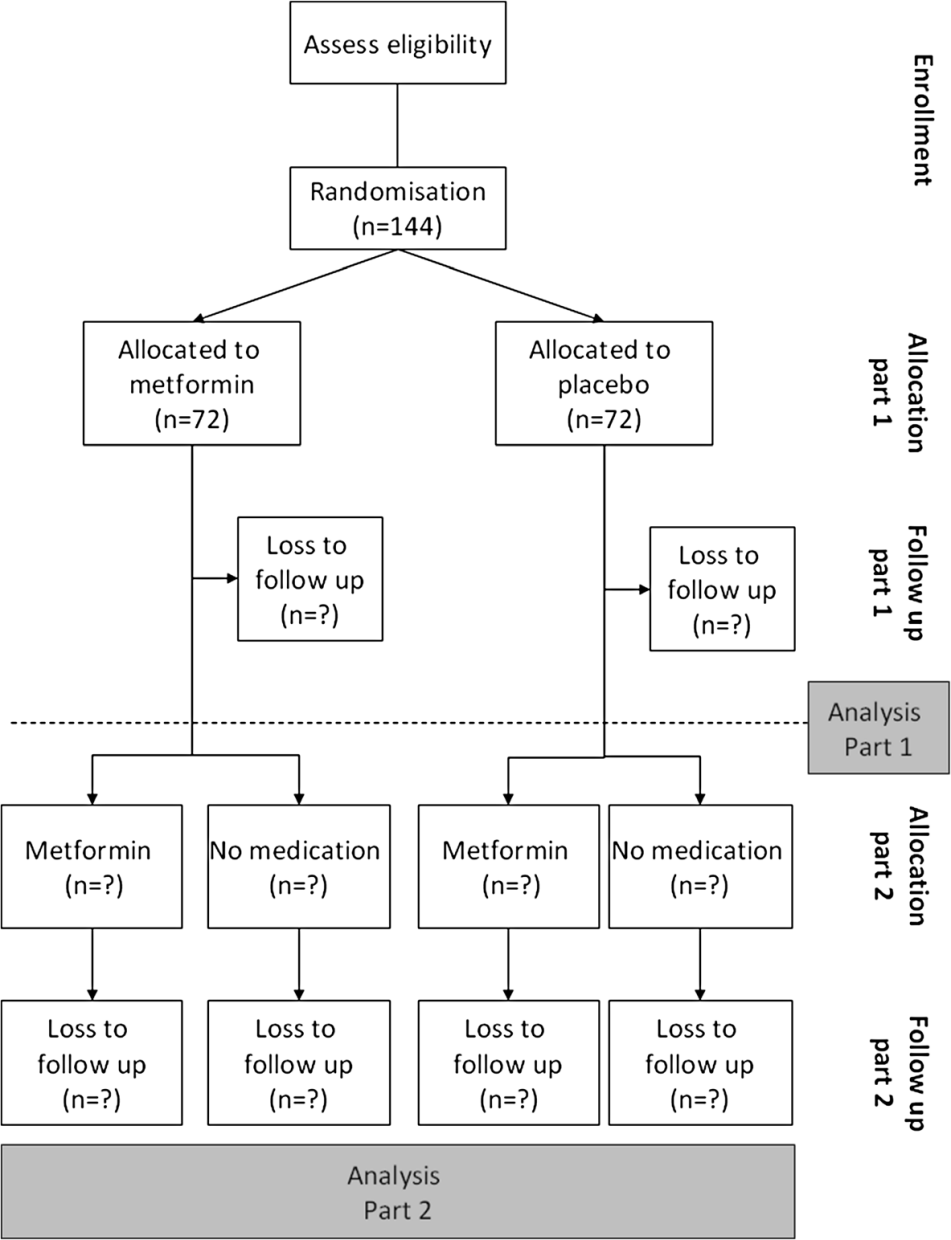
METFORMIN study flowchart.

The metformin study is a multicentre study, divided in two parts, both of 18 months. The first part is a randomized, double-blind placebo controlled trial, with two parallel groups. At study entry, participants are randomized to metformin or placebo for 18 months. All participants will be offered a lifestyle intervention program. This program consists of supervised physical training twice weekly, and individual dietary advice during hospital visits. Participants will visit the hospital 9 times during this part of the study. Between these visits, monthly telephone calls are made.The second part of the study is an open label, follow-up study. Participants who remain obese and insulin-resistant at entrance of the second part, can choose between metformin treatment and no medication. Participants who do not meet these criteria, do not use medication in this part of the study. Therefore, after the follow-up study there are four arms: participants with metformin in both parts, metformin in part 1 and no medication in part 2, placebo in part 1 and metformin in part 2, and placebo in part 1 and no medication in part 2 (Figure 1).

During part 2, no supervised physical training is offered. Participants using Metformin will visit the hospital six times, participants not using medication will have three hospital visits and three phone calls. Participants using Metformin are seen more frequently to monitor potential adverse events.

The study protocol has been approved by the Medical Ethical Committee of the St Antonius Hospital, Nieuwegein, the Netherlands. The study is registered in the Clinical Trials register (ClinicalTrials.gov number NCT01487993).

### Participants

Recruitment of participants takes place at the paediatric outpatient clinics of the study centres. Patients are eligible for this study if they meet the inclusion and exclusion criteria as listed in Table 1. Informed consent will be obtained from all participants/parents or caretakers.

**Table 1:** Inclusion and exclusion criteria

**Inclusion criteria**	**Exclusion criteria**
Age: 10–16 years	Presence of T2DM, polycystic ovarian syndrome, or endocrine disorders treated with corticosteroids
Obesity defined as BMI standard deviation score >2.3	Height <-1.3 SD of target height
Insulin resistance defined as HOMA-IR ≥3.4	Syndromal disorders with or without mental retardation
Caucasian descent	Pregnancy
Informed consent signed by parents and participant	Use of anti-hyperglycaemic drugs, ritonavir or ACE-inhibitors
	(History of) alcohol abuse
	Impaired renal or hepatic function (renal function defined as glomerular filtration rate (GFR) <80 ml/min; GFR = 40 × length (cm)/serum creatinine (μmol/l); hepatic function: alanine aminotransferase >150% of normal value for age)
	Insufficient knowledge of Dutch language

### Sample size

A power analysis for reduction in BMI (primary endpoint) and for reduction of insulin resistance calculated by the HOMA-IR has been performed. For BMI, a sample size of 47 participants per group (metformin and placebo) is sufficient to detect a change in BMI of 2.94% with 90% power. Sample size for HOMA-IR was calculated using a simulation based on retrospective data available from our obesity out-patient clinic. Group sample sizes of 60 patients in both groups were found sufficient for the detection of a difference of 1.6 with a significance level of 0.05. To prevent inadequate power due to drop out of participants, 20% more patients will be included. This means a total amount of 144 children and adolescents have to be included in the study.

### Randomisation

Subjects will be assigned to metformin or placebo in accordance with a randomisation schedule generated by the department of Clinical Pharmacy of the St Antonius Hospital, using PASW Statistics 18.0. Randomisation will be done in blocks of 20 subjects assuring a balanced study after each 20 consecutive inclusions per research site. All research staff is blinded for treatment allocation during the time of the study. Randomisation lists will be kept under secured access in the clinical pharmacy department of both participating hospitals.

The research physician assigns included participants to consecutive study numbers, which correlate with the randomisation and medication number.

### Breaking of the study blind

The study blind will be broken after all participants have finished part 1 of the study. In case of emergencies (serious adverse events, suspected unexpected serious adverse reactions), the blind will be broken after consultation of the principal investigator. Subsequently, these events will be reported to the Medical Ethical Committee.

### Intervention

#### Metformin

After randomisation, all participants will receive either metformin 500 mg tablets or identical placebo tablets. Medication is given according to an increasing dosage regimen. In week 1, participants take 1 tablet daily. Every week, dosage increases with 1 tablet, reaching a maximum of 4 tablets in week 4. This maximum dose will be administered till the end of part 1.

In case the participant develops gastro-intestinal symptoms, dosage will be reduced to the last well-tolerated dosage. Participants will be asked to return remaining study medication every visit. Pill counts will be performed by a research assistant.

During the follow-up study, metformin will be administered according to the dosage regimen of part 1.

### Physical training

During part 1 of the study, physical training will be offered in groups, supervised by a physiotherapist. Training sessions will be twice weekly, and last for one hour. The main goals are creating pleasure in physical exercise, improvement of endurance and coordination. Attendance at the training will be recorded.

All participants will perform a standardised fitness test at study entry, halfway part 1 and at the end of parts 1 and 2.

### Statistical analysis

Data will be analysed using IBM SPSS Statistics. Baseline data will be reported as descriptive statistics. Normally distributed data will be reported as mean ± SD and nonparametric data as median (range).

### Efficacy of metformin

To assess the effect of metformin versus placebo, the Students T test will be used to compare means of normally distributed data and the Mann-Whitney U test to compare nonparametric data. The χ^2^ test will be used for dichotomous outcomes (the development of impaired fasted glucose, impaired glucose tolerance, T2DM, and the presence of micro- and macro- vascular complications versus the study groups).General linear models (analysis of repeated measures) or mixed models (if too much data is missing) will be used to determine the therapeutic effect of the drug. After part 2 of the study, comparisons will be made between the four subgroups (metformin in part 1 and part 2; metformin in part 1 and no medication in part 2; placebo in part 1 and metformin in part 2; placebo in part 1 and no medication in part 2) (Figure 1).

### Safety and tolerability

Safety will be reported as the amount of cases in which hepatic and renal functions exceed safety limits. Tolerability will be reported as descriptive statistics of adverse effects in relation with the achieved dosage level.

### Pharmacokinetics

All observed metformin plasma concentrations will be analysed using nonlinear mixed effects modelling to develop a population pharmacokinetics/pharmacodynamics (PK/PD) model. Using this modelling approach, infrequently obtained samples and observations in the clinical situation can be utilized to analyse determinants of variability in drug response [32-34]. In both the population PK (drug concentrations) and PD (efficacy and safety endpoints) models, the influence of age, bodyweight, BMI, percentage of body fat, gender, Tanner stage, and genetic constitution will be evaluated resulting in individualized dosing regimens. In addition to demographic parameters, the influence of genetic variation in the SLC47A1 gene, which may play an important role in the pharmacokinetics of metformin, is studied [35-37]. If other relevant genes are discovered during the metformin study, these genes will also be determined.

### General procedures and measurements

Table 2 shows which measurements are performed during parts 1 and 2 of the study. In Table 3, blood sampling per visit is specified. Participants in both the metformin and placebo group undergo the same procedures and measurements. Two additional measurements, namely indirect calorimetry, a DEXA scan, and an additional physical test, are performed in study participants included at the Jeroen Bosch hospital study site.

**Table 2:** Measurements during parts 1 and 2 of the study

**Visit**	**Measurements**														
	**Adverse events**	**Co-medication**	**History**	**Physical examination**	**Blood sampling**	**Urine sampling**	**OGTT**	**Metformin day-curve**	**Bio-impedance**	**PWV**	**Fitness test**	**IWQOL questionnaire**	**Dietary diary**	**Calorimetry**^**c**^	**DEXA scan**^**c**^
Part 1															
0			x	x^d^	x	x	x		x	x	x	x	x	x	x
1^a^	x	x													
3	x	x			x										
5	x	x			x										
9^a^	x	x													
13	x	x		x	x										
17^a^	x	x													
21^a^	x	x													
25	x	x		x	x										
29^a^	x	x													
33^a^	x	x													
37	x	x		x^d^	x		x	x	x		x	x	x		x
41^a^	x	x													
45^a^	x	x													
49	x	x		x	x										
53^a^	x	x													
57^a^	x	x													
61	x	x		x	x										
65^a^	x	x													
69^a^	x	x													
73	x	x		x^d^	x	x	x		x	x	x	x	x	x	x
Part 2															
86^b^	x	x													
98	x	x		x	x										
110^b^	x	x													
122	x	x		x	x										
134^b^	x	x													
146	x	x		x	x	x	x		x	x	x	x	x		x

IWQOL, Impact of weight on quality of life; OGTT, Oral glucose tolerance test; PWV, Pulse wave velocity; ^a^Phone calls; ^b^Visit for metformin user, phone call for non-user; ^c^Performed in subsample in one of the study centres; ^d^Extensive examination.

**Table 3:** Specification of measurements per blood sample

**Visit**	**Measurements**												
	**Fasted glucose**	**Fasted insulin**	**OGTT (Glucose and insulin at t = 0, 30, 60, 90 and 120’)**	**HbA1c**	**Blood count, indices**	**Urea, creatinine**	**ALAT**	**Lipid profile**	**Vitamin B12**	**CRP**	**DNA**	**Metformin**	**DNA**
Part 1													
0			x	x	x	x	x	x	x	x	x	x	x
3												x	
5												x	
13	x	x				x	x					x	
25	x	x		x	x	x	x		x			x	
37			x			x	x					x*	
49	x	x		x	x	x	x		x			x	
61	x	x				x	x						
73			x	x	x	x	x	x	x	x		x	
Part 2													
98	x	x		x	x	x	x		x				
122	x	x		x	x	x	x		x				
146			x	x	x	x	x	x	x	x			

ALAT, Alanine aminotransferase; CRP, C-reactive protein; HbA1c, Glycated haemoglobin; OGTT, Oral glucose tolerance test.

*Metformin day-curve (baseline sample and five subsequent samples).

### Adverse events and co-medication

During scheduled phone calls and hospital visits, participants and/or their parents will be asked about adverse events (AEs) and use of co-medication during the past week(s). Collected data for AEs are: start date, stop date, description of AE, kind of action taken regarding study medication (continued, dose adjusted or interrupted, permanently discontinued), therapy for AE, severity of AE (mild, moderate, severe, life threatening, death), whether the AE was expected, whether the AE was serious, and whether the AE occurred during study treatment. Collected data for concomitant medication are: start date, stop date, type, and dose of medication, and duration of use (single dose, intermittent dosage, chronic use). For chronically used medication, changes in prescription, i.e., dose and frequency, will be checked.

### History

At the first visit, an extensive history will be taken. Duration of pregnancy, birth weight, neonatal feeding, and the presence of diabetes gravidarum during pregnancy of the participants is questioned. Further, information on diseases, hospital admissions, and use of medication, alcohol, and tobacco is collected. Regarding family history, data on hypertension, obesity, hypercholesterolemia, cardiovascular disease (myocardial infarction, stroke, transient ischemic attack, peripheral arterial occlusions), and diabetes mellitus in first (parents) and second degree (grandparents, brothers, sisters) family members are collected. Level of education of participants and both parents is recorded, as well as height and weight of both parents. Girls will be asked whether and when they had their menarche.

### Physical examination and anthropometrics

Every visit, except at week 3 and 5, anthropometric measurements are performed. These include: height, weight, and waist, hip, neck, and right wrist circumference.

Height will be measured with a digital stadiometer in the St Antonius Hospital, and with an analogue stadiometer at the Jeroen Bosch hospital. Height will be recorded to the nearest 0.1 cm.

Weight is recorded on a digital scale in all study centres, and is recorded to the nearest 0.05 kg. Waist, hip, neck, and right wrist circumferences are measured with the same tape-measure in both centres, by the research physician. All measurements are recorded to the nearest 0.1 cm. Waist circumference is measured at the level of the navel and hip circumference at the level of the anterior superior iliac spine. Neck circumference is measured three times, the smallest circumference is recorded. Circumference of the right wrist is measured at the level of Lister’s tubercle.

Blood pressure and heart rate are measured with subjects in a seated position using a cuff appropriate for the participants’ arm circumference. In both study centres, blood pressure will be measured electronically.

During the visits in weeks 0, 37, 73, and 146, an extended physical examination is performed by the research physician. This examination includes auscultation of heart, lungs, and abdomen, and abdomen palpation. Abnormal findings will be recorded. The skin will be examined for the presence of acanthosis nigricans, striae, acne, and, in girls, hirsutism. For all participants, pubertal stage according to the classification of Tanner will be recorded. In girls, this includes development of the breasts and pubic hair; in boys, stage of pubic hair and testicular volume is estimated.

### Blood sampling

Blood samples will be collected by venapuncture or, in case of an OGTT, by venous cannula during scheduled hospital visits (Table 2). Before venapuncture or insertion of the venous cannula, local anaesthetics will be applied to the skin (Xylocaine spray, 100 mg/mL, AstraZeneca bv). The specification of measurements per blood sample is shown in Table 3. All samples will be collected by research staff and analyzed in the clinical laboratory of the St Antonius Hospital.

### Urine sampling

Urine samples will be collected at three time points. The sample will be analysed for the concentration of creatinine and micro-albumin, and tested for protein. Additionally, in the first urine sample of female participants, a pregnancy test is performed.

### Oral glucose tolerance test

Oral glucose tolerance tests (OGTT) will be performed at four time points. Participants will come to the hospital after an overnight fast. After insertion of a venous cannula and after obtaining the baseline blood sample (t = 0), participants will receive a solution of glucose: 1.75 g/kg body weight with a maximum of 75 g, dissolved in 200 to 300 mL of water. Blood samples will be taken for glucose and insulin concentrations at 30, 60, 90, and 120 minutes after ingestion of the glucose solution.

### Metformin day-curve

During the OGTT of week 37, a metformin day-curve will be performed. A baseline sample is collected after insertion of the venous cannula (t = 0). Participants will take the oral study medication after ingestion of the glucose solution for the OGTT. Afterwards, samples for serum concentration of metformin are taken at 60, 120, 240, 360, and 480 minutes. Samples will be stored in the toxicological laboratory of the St Antonius Hospital at -20°C until analysis is to be performed.

### Body composition

In order to obtain data on body composition, bio-impedance analysis is performed at regular time-points. In all participating hospitals the same leg-to-leg bio-impedance analysis measurement will be performed using a Tanita BC-420MA body composition analyser (Tanita Corporation, Tokyo, Japan). Participants will stand barefoot on the metal plates of the scale. A correction for weight of clothes (1 kg), sex, age, and height are entered manually into the Tanita system. After analysis, a print out with body weight, estimated percentage of body fat, fat free mass, and total body water is made.

In study participants of the Jeroen Bosch Hospital, a DEXA scan will be performed additionally. The DEXA scan will show the fat distribution and will give the amount of lean and total body mass.

### Arterial stiffness

Arterial stiffness is assessed by pulse wave velocity and augmentation index. The pulse wave velocity measurement is a non-invasive measurement using ultrasound and is measured using the SphygmoCor (Model SCOR-Px, Software version, 7.01; AtCor Medical Pvt. Ltd, Sydney, Australia). Both pulse wave velocity, which correlates with stiffness of the aorta, and augmentation index, reflecting endothelial function, will be used to monitor the development of vascular complications. The measurements will be performed with participants in a supine position. Before and after the measurement, blood pressure will be recorded. All pulse wave velocity measurements will be performed twice. The augmentation index will be calculated in a pulse wave analysis measurement from the right radial artery. For calculation of the pulse wave velocity, the distance between the sternal notch and the expected site for recording at the right carotid artery and right femoral artery will be measured with a tape measure to the nearest 0.1 cm. This distance is entered into the computer before measurements are performed.

### Fitness test

Physical fitness of the participants will be monitored using the modified Shuttle walking test for endurance, while static and dynamic balance tests, according to Movement-ABC will be used to test coordination and strength. All tests will be performed by trained physiotherapists.

### Quality of life

Body weight-related quality of life is measured using the Impact of Weight on Quality of Life-Kids (IWQOL-Kids) questionnaire [38]. This questionnaire consists of 27 items in four domains: physical comfort, body esteem, social life, and family relations; a validated Dutch translation is used [39]. The questionnaire will be handed out during the OGTT, or sent to the participants’ home address to be filled in advance. Participants are free to choose whether they answer the questionnaire in the hospital or at home.

### Diet

Participants will be asked to complete a 3-day dietary diary three times during part 1 of the study, and once during the second part of the study. During the following visit, the diary will be discussed with participants. Advice on a healthy diet is given by the research physician based on this diary. If diet advice is not sufficient a dietician will be consulted.

### Measurements performed in a subsample

#### Indirect calorimetry

This additional test will be performed in one of the participating study centres (Jeroen Bosch Hospital). All of the metabolic processes that occur in the body result ultimately in the production of heat. Direct calorimetry measures the heat production directly; indirect calorimetry makes use of the assumption that all energy-releasing reactions in the body ultimately depend on the utilization of oxygen. Indirect calorimetry, using the ventilated hood principle, is a measurement in which the subject has to lay down in a thermoneutral environment after an overnight fast. In a time span of 15 min, the subject needs to lay still while oxygen uptake and dioxide production of the body are measured. This measurement is an estimate of basal metabolic rate. Calorimetry will be performed when the subject is in fastened condition.

#### DEXA scan

In participants included at the Jeroen Bosch hospital, an additional DEXA scan will be performed. This scan will show fat distribution and will accurately determine lean and total body mass.

#### VO2-max test

Maximal oxygen uptake will be measured using a standardized test for maximal aerobic power: the VO2 max test. The VO2 max test is a single continuous 3- to 5-minute submaximal effort test on a stationary bicycle. It consists of progressive increments in effort (graded exercise) to the point at which the subject will no longer continue to exercise.

### Outcome measures

Along with the multiple objectives in this study, there are multiple outcome measures.

### Primary endpoints and measures

Primary study endpoint for efficacy of metformin is the reduction in BMI standard deviation score, which is calculated from the anthropometric measurements, and the reduction of insulin resistance calculated by the HOMA-IR.

### Secondary outcome measures

Secondary outcome parameters for safety of metformin treatment are hepatic and renal function tests, and concentration of vitamin B12. For tolerability, the number of adverse events (in relation to the achieved dose level) is the outcome parameter.

### Tertiary outcome measures

Tertiary outcome parameters are the PK parameters of metformin in obese children and adolescents. These parameters are estimated using population PK-PD modelling techniques in which a comprehensive covariate analysis will be performed allowing to account for variability in PK parameters on the basis of individual characteristics such as age, bodyweight, BMI, percentage of body fat, gender, Tanner stage, and genetic constitution.

### Quaternary outcome measures

Quaternary outcome parameters for long-term efficacy and long-term safety and tolerability (36 months) of metformin are similar to the primary and secondary outcome measures. In addition, the percentage of patients that has developed impaired fasting glucose, impaired glucose tolerance, and T2DM is evaluated. Furthermore, the development of micro- and macro-vascular complications is assessed, by measuring micro-albuminuria and arterial stiffness.

### Other outcome measures

Other outcome parameters are values of body fat measured by bio-impedance compared to values of body fat measured using DEXA scan, insulin sensitivity measured by the whole body insulin sensitivity index compared with insulin sensitivity calculated by HOMA-IR, HbA1c, β-cell function calculated by HOMA-β%, oral disposition index, insulin secretion calculated by the insulinogenic index, physical fitness measured by validated fitness tests, basal metabolic rate measured by calorimetry, and quality of life measured by validated quality of life questionnaire.

### Limitations of the study

The major limitation of our study is in the open-label, second part of the study. In this part, participants who are still eligible for receiving metformin, i.e., who remain insulin resistant and obese, may choose between taking the drug or not. This will provide a bias in the second part of the study, as participants motivated to lose weight are more likely to choose metformin compared to non-motivated participants. Possibly, this will cause an overestimation of the efficacy of metformin during the second part off the study. This will be taken into account when analysing the results of the second part of the study. The open label construction will not influence adverse reactions to the drug, therefore making it possible to draw firm conclusions about the 36-month safety and tolerability of metformin.

### Discussion and trial status

This article provides the detailed study protocol of our metformin study, including all objectives and outcome measures, a description of the intervention and all study procedures. After completion of the study, the gap in knowledge of long-term effects of metformin on body weight can be filled. Safety and tolerability of metformin use up to 36 months will be investigated. Furthermore, pharmacokinetics of metformin in obese children and adolescents will be known.

Currently, the trial is ongoing and recruitment of participants continues. To date, 60 participants have been included, of whom 13 finished the first part of the trial. Results on efficacy, safety, and tolerability of 18 months of metformin treatment in obese children and adolescents with insulin resistance are expected in summer 2015. First results of PK analysis are expected in autumn 2014. Long-term efficacy, safety, and tolerability results are expected early 2017. These findings will be published in international peer reviewed journals.

## Abbreviations

AE(s): Adverse event(s); BMI: Body mass index; DEXA: Dual-energy X-ray absorptiometry; HOMA-β%: Homeostasis model assessment for beta cell function; HOMA-IR: Homeostasis model assessment for insulin resistance; IWQOL: Impact of weight on quality of life; OGTT: Oral glucose tolerance test; PD: Pharmacodynamics; PK: Pharmacokinetics; T2DM: Type 2 diabetes mellitus.

## Competing interests

None of the authors reports a conflict of interests.

## Authors’ contributions

MV, CK and EM designed the study and secured the funding. MA and ME implemented the study and are responsible for data collection. Both MA and ME contributed equally in drafting the manuscript. MV, CK and EM reviewed and edited the manuscript. All authors take full responsibility for the contents of the manuscript. All authors read and approved the final manuscript.
